# What Do Consumers Think About Foods Processed by Ultraviolet Radiation and Ultrasound?

**DOI:** 10.3390/foods11030434

**Published:** 2022-02-01

**Authors:** Maria Lúcia G. Monteiro, Rosires Deliza, Eliane T. Mársico, Marcela de Alcantara, Isabele P. L. de Castro, Carlos A. Conte-Junior

**Affiliations:** 1 Center for Food Analysis (NAL), Technological Development Support Laboratory (LADETEC), Federal University of Rio de Janeiro (UFRJ), Cidade Universitária, Rio de Janeiro 21941-598, Brazil; conte@iq.ufrj.br; 2 Graduate Program in Food Science (PPGCAL), Institute of Chemistry (IQ), Federal University of Rio de Janeiro (UFRJ), Cidade Universitária, Rio de Janeiro 21941-909, Brazil; isabellepaesleme@gmail.com; 3 Graduate Program in Veterinary Hygiene (PPGHV), Faculty of Veterinary Medicine, Fluminense Federal University (UFF), Niterói, Rio de Janeiro 24220-000, Brazil; elimarsico@gmail.com; 4 Embrapa Food Technology, Rio de Janeiro 23020-470, Brazil; rosires.deliza@embrapa.br; 5 CNPq-PDJ/Embrapa Food Technology, Rio de Janeiro 23020-470, Brazil; marceladealcantara@gmail.com

**Keywords:** emerging non-thermal technologies, novel technologies, UV light, high-intensity ultrasound, free word association, consumer perception

## Abstract

This study aimed to investigate Brazilian consumers’ perception concerning foods processed by ultraviolet (UV) radiation and ultrasound (US) and define consumer segments considering their socioeconomic characteristics and eating habits towards industrialized products through free word association tasks answered by 1004 participants via an online platform. UV- and US-treated foods were more frequently related to unfamiliar words/terms (21.51 and 36.95%) and negative associations (36.25 and 26.70%) than positive ones (29.89 and 24.20%), respectively. Unfamiliarity and health risk concerns were more reported for US-treated foods by women aged 18–25 and ≥46 with low income, and low and frequent industrialized products consumption, as well as for UV-treated foods by consumers ≤35 years old with low and medium income, and low and frequent industrialized products consumption. This indicates that more clear and trustworthy information is needed before introducing these products in the Brazilian market, mainly for potential target consumer groups identified in this study.

## 1. Introduction

Studies concerning effective, eco-friendly, and cost-effective novel processing technologies have been boosted by increasing demand for high-quality, safe, and sustainable foods with no chemical preservatives by consumers together with the global market economic competitiveness (e.g., energy consumption reduction). Innovative food technologies have been highlighted by their effectiveness in several processing applications (tenderizing, brining, emulsification, freezing, thawing, drying, maturation, fermentation, cooking, microbial inactivation, and extension of shelf life), and by their advantages (reduced processing time, energy saving, similar or reduced cost, no or minimal food changes) compared to conventional technologies [1,2,3,4,5]. UV radiation and US are among the most promising novel non-thermal food technologies.

Considering all UV light ranges emitting energy (UV-A: 315–400 nm; UV-B: 280–315 nm; UV-C: 200–280 nm; vacuum UV: 100–200 nm), UV-C is the germicidal range, forming cross-linking between cytosine and thymine (direct action) and producing free radicals from water radiolysis (indirect action) damaging the microbial DNA structure [2]. Among UV-C ranges, the light at 253.7 nm is recognized as an effective and safe technology for food decontamination [6], being the most effective against microorganisms because, in this wavelength, there is the highest light absorption by microbial nucleic acids [2]. The action mechanism of ultrasound (US) at a high intensity (20–100 kHz and 10–1000 W/cm^2^) is based on the cavitation process. The ultrasonic waves are propagated in a liquid medium, generating bubbles, which collapse due to high energy, temperature, and pressure, and create microchannels on foods, leading to free radicals formation and subsequent microbial DNA damage [1].

UV radiation and US are considered cost-effective technologies with easy-to-use installation and operations that do not induce radioactivity or produce toxicity into foods, and may preserve the original food quality when adequately applied [1,3]. Nevertheless, these technologies can generate free radicals causing adverse changes in foods depending mainly on treatment conditions, food composition, and processing [7,8], making it challenging to find an optimal application for each food. At present, identifying the ideal treatment conditions for each food product is the main barrier for using UV radiation and US on an industrial scale. Therefore, although several technical benefits can be obtained for the food production chain by using UV radiation and US, as already stated, food-specific treatment conditions need to be addressed through research for further application of these technologies on a large industrial scale according to the Food and Drug Administration [6,9].

Although scientists know the benefits and risks of foods treated with novel technologies, consumers know little about them [10], especially when they are not commercially available as UV- and US-treated foods. According to some authors, the lack of knowledge or experience with food technological innovations spontaneously evokes doubtful and negative concepts, mainly if the name of the technology may generate misconceptions in interpretation [11,12,13,14]. Furthermore, consumers are increasingly health conscious and aware of the environment [15] and thus have negative associations with industrial processing [12]. In other words, processed foods are being related to unhealthy foods by consumers, which impair the introduction of novel processed products in the marketplace.

Furthermore, socioeconomic, demographic, and eating characteristics also influence the acceptance of novel food technologies [16,17,18,19]. In this context, understanding consumers’ perceptions is crucial to aid future marketing strategies for foods processed by UV radiation and US and guarantee their successful marketability because they are influenced strongly by marketing [18]. Therefore, understanding how consumers perceive foods processed by these two technologies is as important as finding optimal treatment conditions.

The free word association is a projective method with no right and wrong answers. Thus, participants are expected to evoke spontaneous thoughts, feelings, and attitudes [20], which is crucial to understanding their food choices and purchase decisions. This method has been used successfully to evaluate consumers’ perceptions towards conventional products and those processed by innovative technologies such as pulsed electric fields and high hydrostatic pressure [13,14,18]. Despite that, there is no study evaluating the intuitive perceptions of the Brazilian consumers concerning UV- and US-treated foods through a free word association task.

Therefore, this study aimed to understand how foods submitted to UV radiation and US are perceived by Brazilian consumers and identify the associations of consumer segments that would lead to a rejection based on their socioeconomic characteristics and eating habits related to industrialized products.

## 2. Materials and Methods

### 2.1. Participants

The only criteria used in this study for recruiting participants were their interest and availability to collaborate. This study was conducted with a convenient sample to explore overall associations concerning food processed by ultraviolet radiation and food processed by ultrasound for Brazilian consumers and provide valuable qualitative inferences about this subject, with no intent to reflect a specific market population. A total of 1004 Brazilian consumers answered an online questionnaire, including socioeconomic characteristics (Table 1). According to the Brazilian Institute of Geography and Statistics, it is a representative sample of the Brazilian population in terms of age and income [21].

### 2.2. Procedures

This study was approved by the Brazilian Committee of Ethics in Research (CAAE 82850718.2.0000.5243). The questionnaire was developed through a web interface (Google Forms^®^), and the link was distributed via e-mail and social media postings between April and August 2019. The online questionnaire was divided into three sections. In the first and second ones, participants had to write down the first four words, terms, or phrases that came to their minds when they thought about food processed by ultraviolet radiation and food processed by ultrasound, respectively. In the third one, participants were asked to answer five multiple-choice questions: four concerning socioeconomic characteristics (gender, age, income, and career) and one about their consumption frequency of industrialized products (Table 1). All participants were asked to consent to participate in the study before starting it.

### 2.3. Data Analysis

All words, terms, or phrases were considered for data analyses, and their frequency of mentions was calculated separately for UV and US tasks. Associations with similar meanings were sorted into categories and further grouped in dimensions through inductive coding by triangulation. This step was performed and revised by three researchers familiar with the methodology and the final categories and dimensions defined by consensus [13]. A cut-off point was applied to avoid losing a high amount of information. Therefore, the dimensions and categories were considered for analysis when their terms were mentioned by 5% or more of the participants.

The frequency of mention of the categories and dimensions was calculated, and chi-square was applied to identify statistical differences among them considering their different socioeconomic characteristics and consumption frequency of industrialized products. The source of the global chi-square variation was determined using a chi-square per cell test [22]. Correspondence Analysis (CA) was applied in the frequency of mention of the categories, socioeconomic characteristics, and consumption frequency of industrialized products. The principal coordinates (F1 and F2; cumulative inertia above 74%) obtained from CA were submitted to Hierarchical Cluster Analysis (HCA) with the Euclidean distance and Ward’s method. All statistical analyses were carried out at a 5% significance level using the XLSTAT software.

## 3. Results and Discussion

### 3.1. Consumers’ Associations with Food Processed by UV Radiation and Ultrasound

For the word association task concerning UV radiation, participants generated 2554 words, terms, or phrases, of which 436 were different, resulting in an average value of 2.54 associations per participant. A similar pattern was observed for US task (a total of 2503 words, terms, or phrases, of which 471 were different, and an average value of 2.49 associations per participant). These findings corroborate with those found by Guerrero et al. [13], which suggested a clear mental formation of words with an average value of 2.42 associations per participant.

**Figure 1:**
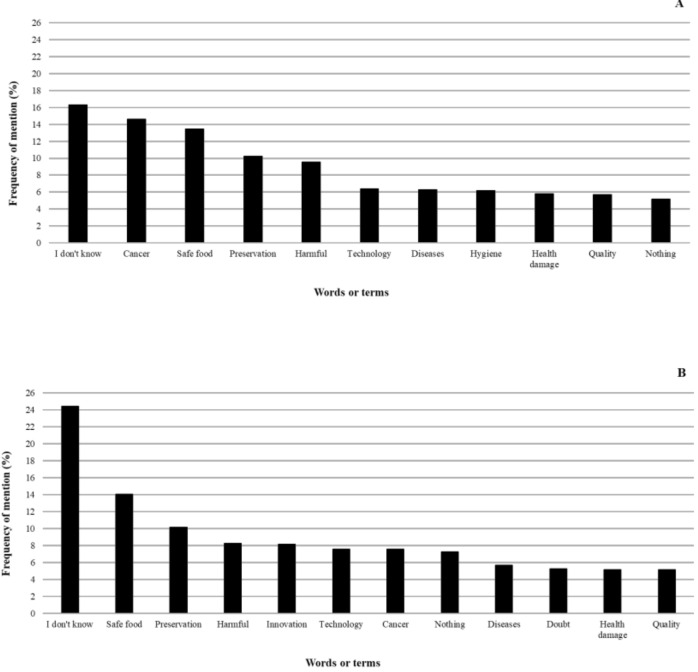
Frequency of mention of the most frequently mentioned words or terms when participants were asked to write down the first four words, terms, or phrases that came to their minds when they thought about food processed by ultraviolet radiation (**A**) and food processed by ultrasound (**B**).

The words or terms most frequently mentioned for UV radiation and ultrasound tasks are shown in Figure 1A,B, respectively.

Among these words or terms, *I don’t know* was the most mentioned term for both technologies indicating that, in general, participants had low knowledge about them. *Doubt* showed a high frequency of mention only in the US word association task, revealing lesser knowledge about US-treated foods than UV-treated ones since the word doubt can also be associated with an unknown/unfamiliar concept. Despite this, *Safe food* also had many mentions for both technologies, which may suggest a controversial feeling considering the citation for *Cancer* (higher for UV). *Quality*, *technology*, and *innovation* were also frequently cited for both technologies. These words have no obvious interpretation and may be related to neutral, positive, or negative associations depending on the consumers’ knowledge concerning benefits and risks from novel technologies.

Overall, although UV radiation and ultrasound evoked positive words/terms (30 and 24%), they elicited more negative ones (36 and 27%), respectively. Furthermore, ultrasound aroused more words/terms associated with unknown/unfamiliar concepts (37%) and non-obvious interpretation (21%) than UV radiation (21 and 12%, respectively). The lack of knowledge regarding intrinsic and extrinsic benefits from innovative technologies applied to foods [12,13] associated with their terminology can scare consumers leading naturally to doubts and negative associations [11,13,14]. In our study, the word radiation and prefix “ultra” may have been determinants for evoking negative words/terms because they can refer to radioactivity and ultra-processed foods, respectively, which are associated with unhealthfulness [23,24].

Consumers’ responses were grouped into categories and dimensions. The frequency of mention of the dimensions and categories for the two technologies and examples of individual responses are presented in Table 2.

The dimensions Negative association, Unfamiliarity, and Negative effects had a high frequency of mention for UV- and US-treated foods. Unfamiliarity, associated mainly with unknown and doubt, was the highest for US, while negative associations such as harmful, unhealthy, bad, disgusting, unsafe, and negative effects associated mainly with cancer were higher for UV than US-treated foods. Within the dimension Negative association, the categories negative hedonic reactions (I wouldn’t buy, I wouldn’t eat) and changes (food changes, oxidation, loss of quality properties) were mentioned only for UV and US, respectively. Dittgen et al. [25] reported that consumers would not buy brown rice treated with UV-C at 4.11 J/cm^2^. Concerning the US, Yildiz et al. [5] observed positive sensory changes applying 100 kW/m^3^ for 15 min. These authors found that the US reduced the browning and decay of fresh-cut quince fruit.

There is no available UV- and US-treated food on the Brazilian market. Nevertheless, UV has been used in food industries to disinfect water, surfaces, surgical supplies, and packaging materials [2]. US is not used industrially in other materials, and studies focus more on the pilot scale [1]. This could explain the higher unfamiliarity with US compared to UV radiation. Concerning negative thoughts, the nomenclature is a crucial point towards consumer perceptions. Overall, those that elicit unnatural things impair the acceptance of new foods and food technologies by evoking wrong, limited, and negative associations even with positive points and benefits compared to existing foods and food technologies [11,26]. In addition to the name “radiation” not being associated with natural things, it can elicit fears due to a lack of knowledge about the actual UV source and its benefits in foods. The association with cancer diseases may be explained by unknown benefits of UV in foods, misconceptions between UV and nuclear radiation sources in association with radiophobia [24]. It is well known that direct exposure to UV radiation may cause changes in the DNA methylation profile leading to skin diseases such as cancer [27] and damage to the eyes [28]. Nevertheless, UV radiation does not induce radioactivity in foods and the environment [24,28]. Besides, there is no research concerning cancer diseases from the consumption of UV-treated foods, and UV at approximately 260 nm is considered safe by the Food and Drug Administration [6]. According to Jack and Sanderson [29], misconceptions and lack of knowledge lead to an instinctive aversion to new technologies and subsequent negative associations. For US, negative perceptions can be attributed to high unfamiliarity and the “ultra” prefix, as previously suggested.

*Safety food* was another highly mentioned dimension, mainly for UV, and *safe food* was the category most relevant within this dimension, mostly associated with decontamination and sterilized food. This was an interesting and unexpected finding because it demonstrated that some Brazilian consumers understand the reasons for applying UV in foods. In Brazil, UV radiation systems are used to decontaminate surfaces and surgical materials in the medical field, and this is sometimes shared on TV and social media. However, little information is provided about UV radiation, with no report about its effects on foods, which may have been suppressed by radiophobia, making it difficult to understand the actual benefits of UV-treated foods. Therefore, participants’ knowledge of food areas about technological strategies against overall microorganism contamination may also have contributed to this finding, as shown in Table 3. Likewise, one of the US applications is inactive microorganisms and extends the shelf life of foods, maintaining or improving their quality attributes [1]. However, ultrasound is the name of an imaging exam, which can impair associations regarding its application for improving food safety. This can be reinforced by research, development, and innovation, an interesting dimension that was only highly mentioned in the US word association task. This dimension was represented by category innovation with associations related to novel technology and recent methods. Although several segments of consumers (uninvolved, rational, careless), including those in favor of innovation (adventurous), most of them are conservatives and tend to worry about the quality and safety of foods treated with novel technologies [30,31]. The lack of knowledge of the benefits and risks of emerging food technologies intensifies this [10].

The dimensions *Technology and food processing*, *Functionality*, *Positive associations*, and *Foods* were frequently cited, especially for US. Within the dimension *Technology and food processing,* the category *technology* associated with food technology was almost equally mentioned for UV and US. The category *industrialized*, associated with the industry and industrialized foods, was only cited for UV, while the category *processing* (treatment, drying, industrial processing) was cited for US. The dimension *functionality* was mainly related to food conservation, and it was associated with two technologies. The category *mechanism of action* (associated with cavitation, vibration, agitation) within the dimension *Functionality* was only cited for US. The dimension *Positive association* was represented by *positive points* such as effectiveness and benefits. These results indicate that despite the association between foods processed by UV radiation and cancer, participants recognized UV radiation as a technology applied in industrialized foods to preserve them. Concerning US, most consumers supposed that it is a food processing technology for preservation. Due to high unfamiliarity with US-treated foods, participants related to food areas can be contributed to this finding since drying, cavitation, vibration, and agitation are specific terms. Our results in Table 3 can reinforce it. Furthermore, some consumers may know ultrasound as a technology, but not for food processing [31].

The dimension *Foods* had *fruits and vegetables* as the category most mentioned, and the main associations were banana, strawberry, lettuce, and carrot. Fruits and vegetables are more commonly consumed raw, increasing consumers’ concerns about contamination [32]. Furthermore, studies have been focused on applying emerging technologies to ensure food safety, especially concerning food products potentially related to outbreak-associated illnesses that are not subjected to the heating process before consumption.

Overall, the dimensions *Others, Quality*, *Shelf life*, and *Recommendations* were equally mentioned for the two technologies. *Others* had *economic aspects* as the most mentioned category, associated with cost, industrial profit, and added value foods, suggesting consumers’ concern about the price of UV- and US-treated foods. However, this barrier can be overcome through appropriate communication that may contribute to acceptance. It is well reported that UV radiation requires short treatment times to achieve its antimicrobial effect saving energy [3]. Therefore, UV radiation is considered a cost-effective technology, and it is believed that UV-treated foods have a similar price to non-treated foods. Likewise, studies concerning US application in foods have been increased worldwide because it is considered an effective, eco-friendly, and cost-effective food processing technology [33]. Price is a common concern of consumers towards new foods and technologies; however, acceptance and purchase intention tend to increase when they have a similar cost to conventional ones [24]. Rojas and Saldaña [34] reported that high prices decreased the purchase intention of US-treated guava juice. Otherwise, Delorme et al. [35] observed lower purchase intention for UV-treated sliced Prato cheese even without a price label; however, it was overcome after providing information about the cost-effectiveness of the technology. Within the dimension *Quality*, the categories *hygiene* and *quality* were almost equally mentioned for UV, and only the category quality was cited for US. *Hygiene* was related to cleanliness and sanitization, and *quality* was associated with food quality and quality assurance. These associations may be explained by the same reasons as those used for the *safe food* category. According to Deliza and Ares [12], besides naturalness and price, consumers have habitual concerns about the effects of technologies on food quality and safety. Rojas and Saldaña [34] observed that guava juice treated with ultrasound had lower acceptance than a non-treated one; however, this acceptance was increased after providing information about ultrasound benefits due to consumers’ higher perception concerning quality and safety. The same was observed in sliced Prato cheese processed by UV [35].

The dimensions *Shelf life*, related to increased shelf life, prolonged conservation, and storage, and *Recommendations*, associated with worrying, unfit for consumption, and health risks reinforce our findings concerning safe food and hygiene versus harmful, unhealthy, unsafe, diseases, and the unknown.

It is worth highlighting similarities among dimensions, categories, and associations in the UV and US tasks. However, although UV-treated foods have been more associated with safe food and quality assurance, they were more related to detrimental aspects (unsafe, unhealthy, diseases, cancer), including aversion to buying or eating them, even with a higher lack of knowledge about US-treated foods.

### 3.2. Effects of Socioeconomic Characteristics and Frequency of Consumption of Industrialized Products on Consumers’ Associations

Personal habits are one of the main factors influencing the acceptance of food innovations [36]. In general, individuals with a healthy lifestyle give crucial importance to food–health relationships looking for fresh and natural foods and thus, tend to be disgusted by processed ones [14,18]. Nevertheless, the most differences found were between whether participants were related to food areas or not (Table 3). Obviously, students or professionals of food areas more frequently mentioned the categories *conservation*, *quality*, *fruit and vegetables*, *positive points*, and *shelf life* for UV- and US-treated foods, *hygiene* for UV, and *mechanism of action*, *technology*, *processing*, *safe food*, and *innovation* for US. Otherwise, they less frequently cited *unknown* and *diseases* for UV- and US-treated foods, *bad*, *detrimental*, *negative hedonic reactions*, *technology*, and *industrialized* for UV, and *doubt*, *health damage*, *negative points*, and *recommendations*, *alerts*, *and risks* for US. On the other hand, opposite associations were observed for lay consumers.

The category *unknown* had lower mention by males than females for UV and US (Table 3), indicating a lack of knowledge, especially among women. Similarly, Sajdakowska et al. [37] also reported that Polish women had a higher lack of knowledge about food technologies than men. The categories *diseases, recommendations, alerts, and risks* were more cited for UV-treated foods by consumers aged 18–35 y, while those aged 26–35 y associated US-treated foods more frequently with the words *bad*, *detrimental*, and less frequently with *processing*. The higher proportion of women than men among the participants may be contributed to our finding, representing a limitation of this study. On the other hand, consumers aged ≥46 y mentioned the categories *bad, detrimental,* and *fruits and vegetables* more frequently for UV, and *health damage* for US. These findings demonstrate the role of age, indicating that older consumers were more negative and concerned about novel food technologies. Overall, Brazilians above 40 years old eat more frequently healthy foods, including fruits and vegetables [16], which are unprocessed or minimally processed foods. Moreover, older consumers care more about the safety and familiarity regarding food products [19]. According to Zheng et al. [38], the risk perception towards health tends to increase as people get older.

The category *safe food* was less cited by consumers with income between 1–5 Brazilian minimum wage (BMW), but *diseases* received more mentions for the same income about UV-treated foods. An opposite finding was found for consumers with income >20 BMW. The categories *industrialized, recommendations, alerts, and risks* had more mentions from consumers with income >5–10 BMW (Table 3). Vidigal et al. [19] reported that lower-income individuals had a lower acceptance of novel food technologies. They attributed it to a higher lack of knowledge of this group of individuals, similarly to the present study. The greater concern of the low-income consumers may be due to less access to information from education, public and online media than medium- and high-income ones. Furthermore, low-income individuals are more afraid of getting sick because they have difficulty accessing and receiving quality healthcare services [39]. Otherwise, the medium-income consumers (>5–10 BMW) demonstrated a greater concern about health risks from US-treated foods than low-income ones. Consumers with income >5–10 BMW more frequently cited the category *innovation*. Despite that, US-treated foods were more associated with *bad, detrimental*, and less related to *conservation* by consumers with income >5–10 BMW. These findings may be attributed to higher unfamiliarity for US-treated foods (about 45%) than UV-treated ones (little over 25%). Furthermore, foods processed by US (36.95%) evoked more words/terms associated with unknown/unfamiliar concepts than foods processed by UV radiation (21.51%). According to some authors, the lack of knowledge concerning benefits and risks from novel food technologies spontaneously evokes doubts and negative associations [11,13,14]. The application of US in foods is still evaluated in pilot-scale experiments by the scientific community [1], and unlike UV radiation, US is not used commercially in other materials. Therefore, there is no information for all lay populations regarding the use of US as a decontamination processing.

The category *bad, detrimental* was more cited for UV-treated foods, and *health damage* for US-treated foods by consumers with low consumption frequency of industrialized products, while less cited by consumers with high consumption frequency of industrialized products. Consumers with a low consumption frequency of industrialized products also felt more negative hedonic reactions towards UV-treated foods (Table 3).

### 3.3. Consumer Groups about Foods Processed by UV Radiation and US Considering Socioeconomic Characteristics and Consumption Frequency of Industrialized Products through Chemometric Analysis

**Figure 2:**
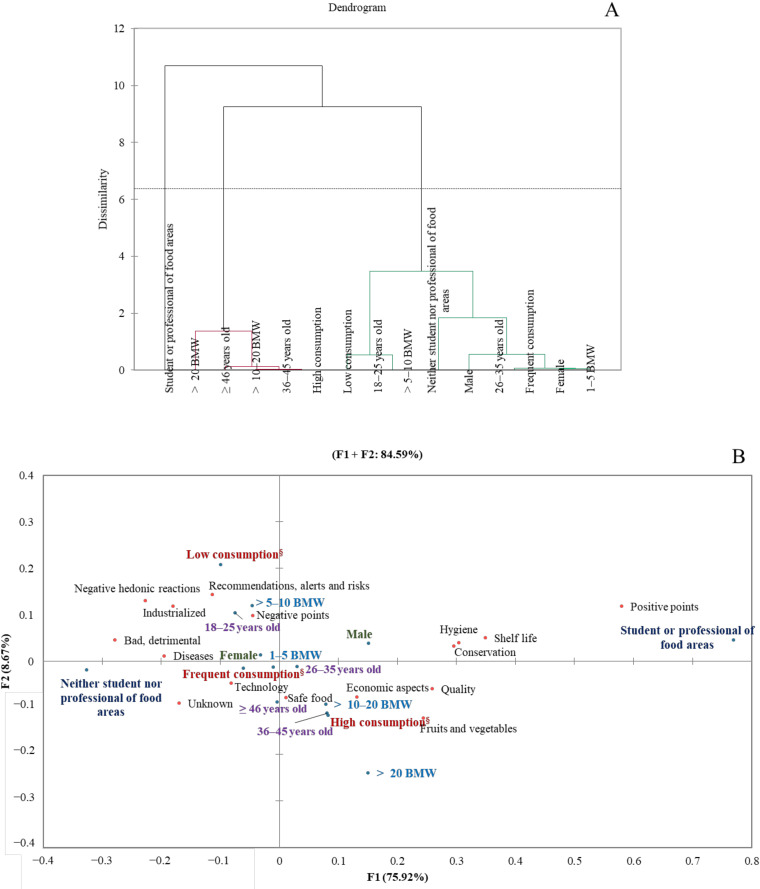
Hierarchical cluster analysis (**A**) and correspondence analysis of the consumer characteristics (blue) and frequency of mention of the categories (red) from free word association task concerning ultraviolet (UV)-treated foods (**B**) at 5% significance level. BMW—Brazilian minimum wage in Brazilian currency (Real). Low consumption—consumers who never or rarely consume industrialized products; frequent consumption—consumers who consume frequently industrialized products; high consumption—consumers who consume daily industrialized products or more than once a day.

Three consumer groups were formed concerning consumer perception of food products processed by UV radiation (Figure 2A). Cluster 1 included male and female consumers aged 18–35, with income ranging from 1 to 10 BMW, neither students nor professionals of food areas, and low and frequent consumption of industrialized products. This consumer group associated UV-treated foods with bad, detrimental, negative points, negative hedonic reactions, unknown, diseases, recommendations, alerts and risks, technology, industrialized, and safe food (Figure 2B). Cluster 2 had consumers aged 36 or more with income above 10 BMW and high consumption of industrialized products (Figure 2A). This consumer group related UV-treated foods with safe food, economic aspects, technology, quality, fruits and vegetables, unknown, and diseases (Figure 2B). The students or professionals of food areas belonged to cluster 3 (Figure 2A) and associated UV-treated foods with safe food, conservation, hygiene, positive points, and shelf life (Figure 2B). Based on our findings, lay and younger consumers (≤35 years old) with low and medium-income (1–10 BMW) who consume industrialized products frequently, rarely, or never, were the most concerned about the adverse effects of UV-treated foods. However, they recognized UV radiation as an industrial strategy to ensure safe food. Older consumers (≥36 years old) with high income (>10 BMW) who consume industrialized products daily or more than once a day perceived UV-treated foods as safe food. Nevertheless, this consumer group also demonstrated unfamiliarity and concerns about price and diseases from food products processed by UV radiation. Our results indicate a lack of knowledge and health risk concerns towards UV-treated foods regardless of gender, age, income, and eating habits related to industrialized products consumption. However, it was more remarkable in the consumer group comprised of lay and younger (≤35 years old) consumers with low and medium-income (1–10 BMW) who consume industrialized products frequently, rarely, or never.

**Figure 3:**
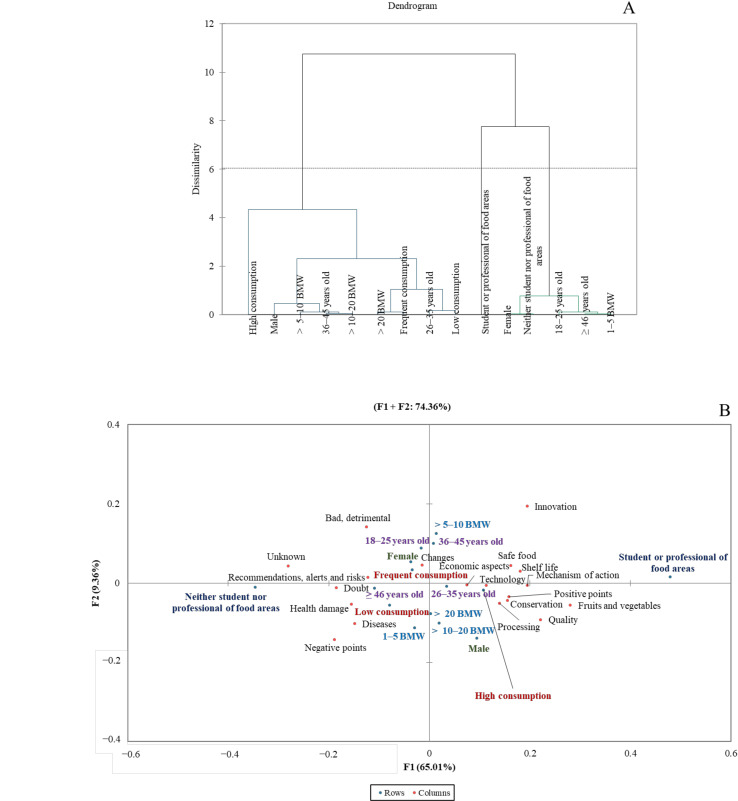
Hierarchical cluster analysis (**A**) and correspondence analysis of the consumer characteristics (blue) and frequency of mention of the categories (red) from free word association task concerning ultrasound (US)- treated foods (**B**) at 5% significance level. BMW—Brazilian minimum wage in Brazilian currency (Real). Low consumption—consumers who never or rarely consume industrialized products; frequent consumption—consumers who consume frequently industrialized products; high consumption—consumers who consume daily industrialized products or more than once a day.

Three consumer groups were identified regarding consumer perception of food products processed by US (Figure 3A). Cluster 1 comprised males aged 26–45 with income >5 BMW and different consumption frequencies of industrialized products (low, frequent, and high). This consumer group associated US-treated foods with processing, technology, conservation, positive points, safe food, shelf life, quality, and economic aspects (Figure 3B). This consumer group was also unfamiliar and concerned about negative issues and health risks from foods processed by US. However, it was reported by consumers with low and frequent consumption of industrialized products according to CA analysis since consumption frequency of industrialized products was not determinant to consumer segmentation by HCA analysis. It occurred possibly because almost half of the consumers were unfamiliar with US-treated foods.

Cluster 2 included females aged 18–25 and ≥46 with income from 1 to 5 BMW, neither student nor professional of food areas (Figure 3A). US-treated foods were related to unknown, doubt, changes, recommendations, alerts and risks, bad, detrimental, negative points, health damage, and diseases by these consumers (Figure 3B). Cluster 3 was composed of students or professionals of food areas (Figure 3A). As expected, this consumer group was more optimistic about US-treated foods and associated it with conservation, mechanism of action, technology, positive points, safe food, innovation, and shelf life (Figure 3B).

Overall, consumers do not know about novel food technologies and tend to make negative associations because they cannot perceive their benefits and risks [10]. Despite this, previous studies have reported different associations for novel food technologies depending on gender, age, income, and eating habits [16,17,18,19]. In general, women are more related to household food activities, resulting in a greater concern regarding proper foods for the family than men [40]. Furthermore, as women tend to have healthier eating habits, they are less willing to accept novel food technologies than men [16,41]. Similar to our findings, previous studies also reported that women perceived novel food processing technologies more negatively than men [18,37]. Concerning age, most studies show that younger adults are more accepting of novel food technologies than older ones [18,19,36]. Older adults have deep-rooted traditional habits, more cautious behavior seeking familiar products, and more interest in maintaining health and preventing chronic diseases [19,30]. According to Garrido and Gallardo [17], older adults (53–71 years old) are less likely to be convenient and eat less processed food because they like to cook and consider it healthier. However, in our study, younger adults (18–25 years old) also had negative perceptions and were concerned about the health risks of US-treated foods. This can be attributed to information and knowledge sharing in online media, which strongly contribute to increasing the number of health-conscious consumers, mainly young adults who spend more time connected.

Regarding income, a higher lack of knowledge due to limited access to information and a greater fear of getting sick due to limited access to a quality healthcare service are reasons frequently used to explain the lower acceptance of novel food technologies by lower-income consumers. Martins et al. [18], Vidigal et al. [19], and Young et al. [36] also observed that lower-income individuals were less accepting of innovative food technologies than medium- and high-income ones corroborating with our findings.

Regarding eating habits, consumers with low and frequent consumption of industrialized products perceived US-treated foods more negatively than consumers with high consumption of industrialized products. Consumers with healthy eating habits consume less processed foods and, thus, are less likely to accept novel food technologies [14,17,18]. The negative perceptions concerning foods processed by US from consumers with frequent consumption of industrialized products may be attributed to unfamiliarity with this type of food technology and its prefix “ultra”. The lack of knowledge of the benefits and risks of novel food technologies and their terminologies can result in doubts and spontaneous negative associations [11,12,13,14]. The prefix “ultra” may have been associated with industrial processing and, thus, with unnatural food. Martins et al. [18] observed that consumers perceived cold-pressed juice more positively than pressurized juice and pasteurized juice and attributed it to terminology, wherein the words “pressed” and “cold may have evoked associations with unprocessed and natural products.

Roselli et al. [42] reported that almost half of the participants (49%) would not buy and the other half ones would buy (51%) extra-virgin olive oil extracted by ultrasound considering a sample population of 54% women and 68% individuals with high income. Otherwise, based on a population composed of 72% women and young individuals (19–33 years), Rojas and Saldaña [34] observed low acceptance and purchase intention for guava juice processed by US, which only was improved after proving information label about higher stability and nutrients absorption.

Concerning UV-treated foods, no clear segmentation was observed for lay consumers. However, those youngers with low and medium income and with low and frequent consumption of industrialized products were more pessimistic. All consumers perceived UV-treated foods as safe food; however, they also demonstrated health risk concerns. Similarly, Jack and Sanderson [29] reported that most consumers perceived benefits from food irradiation, but few of them were willing to buy irradiated food and attributed it mainly to radiophobia. Overall, consumers still associate radiation with radioactivity and nuclear energy [24]. Lima Filho et al. [43] reported that Brazilian consumers related food irradiation with a preservation method (31.4%) and reduction of microorganisms (22.5%); however, they associated more negatively irradiated foods with radioactivity (52.8%), cancer (48.7%), nuclear accident (27.2%), and mutation (19.6%), reinforcing our findings. A recent study concluded that the acceptance of irradiated foods is increased by not expressing the term “radiation” and adding “food safety” as one benefit in the labeling [44]. As Brazilian consumers seem to understand the benefits of UV-treated foods, expressions regarding “no radioactivity” in the labeling may also be needed. Delorme et al. [35] demonstrated that the low acceptance and purchase intention of UV-treated Prato cheese was overcome by label information about the technological advantages such as elimination of microorganisms, minimal changes to food, absence of residues and toxic effects, besides cost and energy effectiveness in comparison with other preservation methods. These authors considered Brazilian consumers composed of 69% women, 70% young individuals (18–35 years), 56% with high education level, and 54% with low–medium income. In 2017, young Brazilian consumers were less concerned about risks from irradiated foods [43]. In our study, younger individuals were more pessimistic, which may be explained by increased health-consciousness among these consumers suggesting that a greater effort will be needed to introduce foods processed by UV in the market.

## 4. Conclusions

Brazilian consumers are unfamiliar and less willing to accept foods processed by UV radiation or US technologies. UV-treated foods were considered safe foods; however, most consumers associated them with unfamiliarity and health risks regardless of gender, age, income, and eating habits related to industrialized products consumption, especially those aged ≤35 years old with low and medium income (1–10 BMW) and low and frequent consumption of industrialized products. The consumers more unfamiliar and concerned about risks from foods processed by US were women aged between 18 and 25 and above 46 years old with low income and low and frequent consumption of industrialized products.

Therefore, more transparent and trustworthy information about the benefits of UV- and US-treated foods must be shared with the overall Brazilian population to avoid wrong interpretations and rejection of these products caused by lack of knowledge and confidence, especially for those specific consumer groups identified in this study. Simultaneously, marketing strategies such as renaming technologies and adding benefits/no risks for the consumers should be considered. Furthermore, younger consumers (from 18 to ≤35 years old) may be a potential target group to avoid future problems introducing novel food technologies in the marketplace.

## Figures and Tables

**Table 1:** Information concerning socioeconomic characteristics and consumption frequency of industrialized products of the participants (*n* = 1004).

	%
Gender	
Female	72.34
Male	27.66
Age (Years Old)	
18–25	32.94
26–35	36.12
36–45	16.32
≥46	14.62
Income—Brazilian minimum wage (BRL 1045.00) ^¥^	
1 to 5	48.36
>5 to 10	26.17
>10 to 20	16.52
>20	8.95
Career ^£^	
Food areas	32.14
Other areas	67.86
Consumption Frequency of Industrialized Products ^€^	
Low	20.59
Frequent	50.65
High	28.76

^¥^ In Brazilian currency (Real). ^£^ Career—food areas (student or professional of food engineering or science, veterinary medicine, pharmacy, and nutrition), and other areas (neither student nor professional of food areas). ^€^ Consumption frequency of industrialized products—low (consumers who never or rarely consume industrialized products), frequent (consumers who frequently consume industrialized products), and high (consumers who consume industrialized products daily or more than once a day).

**Table 2:** Frequency of mention of the dimensions, categories, and examples of individual associations identified in the word association task about food processed by ultraviolet radiation and ultrasound.

Dimensions	Categories (Examples of Associations)	Frequency of Mention (%)	
		Ultraviolet	Ultrasound
*Negative association*		*28*	*24*
	Bad, detrimental (harmful, unhealthy, bad food)	18	6
	Negative points (disgusting, weird, unsafe)	6	5
	Negative hedonic reactions (I wouldn’t buy, I wouldn’t eat)	5	0
	Changes (food changes, oxidation, loss of quality properties)	0	5
*Unfamiliarity*		*27*	*44*
	Unknown (I don’t know, unknown)	20	29
	Doubt (uncertainty, doubt)	0	7
*Safety food*		*22*	*12*
	Safe food (decontamination, safe food, sterilized food)	24	12
*Negative effects*		*19*	*25*
	Diseases (cancer, carcinogenic, tumor)	21	11
	Health damage (harmful to health, toxic, death)	0	12
*Others*		*18*	*18*
	Economic aspects (cost, industrial profit, added value)	6	6
*Technology and food processing*		*17*	*24*
	Technology (technology, food technology)	7	8
	Industrialized (industry, industrialized foods)	6	0
	Processing (treatment, drying, industrial processing)	0	6
*Functionality*		*17*	*27*
	Conservation (food preservation)	11	12
	Mechanism of action (cavitation, vibration, agitation)	0	13
*Quality*		*14*	*13*
	Hygiene (cleanliness, sanitization)	7	0
	Quality (food quality, quality assurance, quality)	6	6
*Foods*		*14*	*22*
	Fruits and vegetables (banana, strawberry, lettuce, carrot)	7	5
*Positive associations*		*9*	*14*
	Positive points (effective, benefits)	6	10
*Shelf life*		*8*	*7*
	Shelf life (increased shelf life, prolonged conservation, storage)	8	7
*Recommendations*		*7*	*9*
	Recommendations, alerts and risks (worry, unfit for consumption, health risks)	7	9
*R&D&I ^¥^*		*0*	*10*
	Innovation (novel technology, recent method)	0	8

^¥^ Research, development, and innovation.

**Table 3:** Frequency of mention of the categories from ultraviolet (UV) radiation and ultrasound (US) word association tasks according to socioeconomic characteristics (gender, age, income, and career) and consumption frequency of industrialized products of the participants.

UV Radiation Task															
Categories	Gender		Age (years old)				Income (BMW) ^¥^				Career ^£^		Consumption Frequency of Industrialized Products ^€^		
	Male	Female	18–25	26–35	36–45	≥46	1–5	>5–10	>10–20	>20	Food Areas	Other Areas	Low	Frequent	High
Bad, detrimental	4.38	13.35	6.18	5.88	2.39	3.29 (+) *	8.76	5.18	2.49	1.29	0.60 (−) ***	17.13 (+) ***	4.68 (+) *	9.46	3.59 (−) **
Negative points	1.89	3.78	2.09	2.09	1.00	0.50	3.19	1.49	0.50	0.50	1.49	4.18	1.49	2.89	1.29
Negative hedonic reactions	1.29	4.08	1.69	2.49	0.60	0.60	2.39	1.89	0.60	0.50	0.50 (−) ***	4.88 (+) ***	1.99 (+) **	2.19	1.20
Unknown	3.98 (−) **	15.84 (+) **	5.88	7.27	3.49	3.19	9.86	4.48	3.78	1.69	2.49 (−) ***	17.33 (+) ***	3.19	10.66	5.98
Safe food	7.17	16.83	7.07	9.96	4.18	2.79	9.86 (−) *	6.08	4.98	3.09 (+) *	6.87	17.13	3.78	12.15	8.07
Diseases	4.78	16.14	8.17 (+) *	8.07	2.59	2.09	11.45 (+) *	5.08	3.39	1.00 (−) *	2.39 (−) ***	18.53 (+) ***	3.78	10.76	6.37
Economic aspects	1.79	3.78	1.69	2.19	1.10	0.60	2.99	0.90	0.90	0.80	2.29	3.29	0.90	2.79	1.89
Technology	2.39	5.28	2.19	3.29	1.10	1.10	3.49	2.09	1.49	0.60	1.49 (−) *	6.18 (+) *	1.39	3.39	2.89
Industrialized	1.99	3.78	2.19	2.49	0.50	0.60	2.19	2.19 (+) *	1.00	0.40	0.70 (−) *	5.08 (+) *	1.39	3.29	1.10
Conservation	3.59	7.77	2.79	5.28	2.19	1.10	5.28	3.09	2.09	0.90	6.77 (+) ***	4.58 (−) ***	2.19	5.78	3.39
Hygiene	2.29	4.48	1.89	2.79	1.10	1.00	2.59	2.09	1.29	0.80	4.08 (+) ***	2.69 (−) ***	1.39	3.49	1.89
Quality	2.59	3.98	1.79	2.49	1.29	1.00	3.19	1.29	1.29	0.80	3.49 (+) ***	3.09 (−) ***	0.90	3.59	2.09
Fruits and vegetables	2.29	4.98	1.59	2.69	1.20	1.79 (+) **	2.99	1.89	1.00	1.39	3.78 (+) ***	3.49 (−) ***	1.00	3.59	2.69
Positive points	2.69	3.39	2.09	2.19	0.90	0.90	2.79	1.39	1.39	0.50	5.38 (+) ***	0.70 (−) ***	1.29	3.09	1.69
Shelf life	2.19	5.98	2.89	3.19	1.10	1.00	4.18	2.09	1.39	0.50	5.48 (+) ***	2.69 (−) ***	1.29	3.78	3.09
Recommendations, alerts and risks	1.89	5.08	3.09 (+) *	2.09	0.90	0.90	3.29	2.59 (+) *	0.80	0.50	1.49	5.48	1.79	3.29	1.89
**US Task**															
**Categories**	**Gender**		**Age (years old)**				**Income (BMW) ^¥^**				**Career ^£^**		**Consumption Frequency of Industrialized Products ^€^**		
	**Male**	**Female**	**18–25**	**26–35**	**36–45**	**≥46**	**1–5**	**>5–10**	**>10–20**	**>20**	**Food areas**	**Other areas**	**Low**	**Frequent**	**High**
Unknown	6.27 (−) **	22.91 (+) **	8.76	11.25	5.08	4.08	13.84	7.37	5.78	2.19	3.29 (−) ***	25.90 (+) ***	5.28	15.84	8.07
Doubt	1.59	5.98	2.39	2.59	1.10	1.49	4.18	1.59	1.29	0.50	1.79 (−) **	5.78 (+) **	1.79	3.78	1.99
Conservation	3.69	8.17	3.78	4.18	2.49	1.39	6.67	2.19 (−) *	1.99	1.00	6.87 (+) ***	4.98 (−) ***	1.79	6.18	3.88
Mechanism of action	3.49	9.46	4.78	5.08	1.69	1.39	6.47	3.29	1.89	1.29	8.27 (+) ***	4.68 (−) ***	2.89	6.08	3.98
Diseases	3.59	8.07	4.68	4.28	0.70	1.99	6.47	2.59	1.29	1.29	2.99 (−) ***	8.67 (+) ***	2.59	5.78	3.29
Health damage	3.78	8.17	3.69	3.49	2.29	2.49 (+) *	5.18	3.59	1.69	1.49	3.19 (−) ***	8.76 (+) ***	3.39 (+) *	6.08	2.49 (−) *
Bad, detrimental	1.39	4.58	1.49	3.09 (+) *	0.50	0.90	2.49	2.29 (+) *	0.90	0.30	1.79	4.18	1.49	3.09	1.39
Negative points	1.59	3.59	2.29	1.10	1.20	0.60	2.59	0.90	1.00	0.70	1.00 (−) ***	4.18 (+) ***	0.80	2.79	1.59
Changes	1.89	3.49	1.79	2.29	0.70	0.60	2.99	1.59	0.50	0.30	2.59	2.79	0.50	3.29	1.59
Technology	2.49	5.98	3.49	2.79	1.10	1.10	3.78	2.59	1.59	0.50	4.48 (+) *	3.98 (−) *	1.59	3.69	3.19
Processing	1.59	4.48	2.59	1.29 (−) *	1.49	0.70	3.39	1.69	0.80	0.20	3.49 (+) *	2.59 (−) *	1.20	3.19	1.69
Fruits and vegetables	2.19	2.89	1.99	1.99	0.60	0.50	2.59	1.39	0.80	0.30	3.49 (+) **	1.59 (−) **	0.50	2.59	1.99
Economic aspects	1.39	4.78	1.59	2.49	1.10	1.00	2.89	1.20	1.39	0.70	2.59	3.59	1.20	3.19	1.79
Positive points	3.69	6.67	2.99	4.38	1.69	1.29	5.28	2.49	1.59	1.00	5.98 (+) ***	4.38 (−) ***	2.19	4.48	3.69
Quality	2.29	3.88	1.99	1.69	1.39	1.10	3.09	1.29	1.29	0.50	3.98 (+) ***	2.19 (−) ***	1.39	2.29	2.49
Safe food	3.29	9.26	3.69	5.38	2.09	1.39	6.08	3.49	1.69	1.29	7.47 (+) ***	5.08 (−) ***	2.09	6.27	4.18
Innovation	1.59	6.87	2.29	3.29	1.59	1.29	2.79	3.09 (+) *	1.99	0.60	5.48 (+) ***	2.99 (−) ***	1.00	4.68	2.79
Recommendations, alerts and risks	2.29	6.47	2.69	3.19	1.29	1.59	4.38	2.99	0.90	0.50	2.59 (−) *	6.18 (+) *	2.19	4.28	2.29
Shelf life	2.09	5.08	2.29	2.59	1.29	1.00	3.39	2.09	1.20	0.50	4.48 (+) ***	2.69 (−) ***	1.39	3.78	1.99

^¥^ Brazilian Minimum wage (BLR 1045.00) in Brazilian currency (Real). ^£^ Formation—food areas (student or professional of food engineering or science, veterinary medicine, pharmacy, and nutrition), and other areas (neither a student nor professional of food areas). ^€^ Consumption frequency of industrialized products—low (consumers who never or rarely consume industrialized products), frequent (consumers who frequently consume industrialized products), and high (consumers who consume daily industrialized products or more than once a day). (+) or (−) indicate that the observed value is higher or lower than the expected theoretical frequency according to the chi-square per cell: * *p* < 0.05; ** *p* < 0.01; *** *p* < 0.001.

## Data Availability

The data presented in this study are available on request from the corresponding author.
